# Calcium-fortified beverage supplementation on body composition in postmenopausal women

**DOI:** 10.1186/1475-2891-4-21

**Published:** 2005-06-21

**Authors:** Mark D Haub, Tammy R Simons, Chad M Cook, Valentina M Remig, Enas K Al-Tamimi, Carol Ann Holcomb

**Affiliations:** 1 Department of Human Nutrition, Kansas State University, Manhattan, KS, USA; 2 Galichia Center on Aging, Kansas State University, Manhattan, KS, USA

**Keywords:** aging, older, ascorbic acid, juice, obesity.

## Abstract

**Background:**

We investigated the effects of a calcium-fortified beverage supplemented over 12 months on body composition in postmenopausal women (n = 37, age = 48–75 y).

**Methods:**

Body composition (total-body percent fat, %Fat_TB_; abdominal percent fat, %Fat_AB_) was measured with dual energy x-ray absorptiometry. After baseline assessments, subjects were randomly assigned to a free-living control group (CTL) or the supplement group (1,125 mg Ca^++^/d, CAL). Dietary intake was assessed with 3-day diet records taken at baseline and 12 months (POST). Physical activity was measured using the Yale Physical Activity Survey.

**Results:**

At 12 months, the dietary calcium to protein ratio in the CAL group (32.3 ± 15.6 mg/g) was greater than the CTL group (15.2 ± 7.5 mg/g). There were no differences from baseline to POST between groups for changes in body weight (CAL = 0.1 ± 3.0 kg; CTL = 0.0 ± 2.9 kg), %Fat_TB _(CAL = 0.0 ± 2.4%; CTL = 0.5 ± 5.4%), %Fat_AB _(CAL = -0.4 ± 8.7%; CTL = 0.6 ± 8.7%), or fat mass (CAL = 1.3 ± 2.6 kg; CTL = 1.3 ± 2.7 kg).

**Conclusion:**

These results indicate that increasing the calcium to protein ratio over two-fold by consuming a calcium-fortified beverage for 12 months did not decrease body weight, body fat, or abdominal fat composition in postmenopausal women.

## Background

According to results and concepts from Zemel et al. [1] and epidemiologic studies [2-4], dietary calcium plays a role in regulating body composition. The epidemiological data demonstrate an inverse association between calcium intake and body weight and body fat mass [2,3,5]. Davies et al. [2] derived a prediction equation using the ratio of intakes of calcium (mg/day) and protein (g/day) (calcium:protein ratio) to estimate annual changes in body weight. The benefit of the calcium:protein ratio is that it tends to correct inaccuracies of self-reported dietary intakes of the individual nutrients [2]. Zemel et al. [1] reported a significant decrease in body fat in African-American males with Type 2 diabetes when they supplemented their diets with 600 mg of calcium from dairy products. In a different randomized clinical trial, Zemel [6] illustrated that those with a higher calcium intake compared to those consuming a calcium deficient (400–500 mg calcium/day) diet had less body fat. While these studies are valuable in demonstrating associations and the effects of deficient diets, there is limited data illustrating the effect of calcium supplementation on body composition in those on self-selected free-living diets. A retrospective study by Shapses et al. [7] observed that calcium supplementation over six months did not increase weight loss or fat loss in postmenopausal women consuming a hypoenergetic diet. Likewise, Barr et al. [8] observed that milk-supplementation (three servings/day) did not elicit weight loss or improve metabolic risk factors in older adults. Given the discrepancy between studies, the effect of calcium intake on body weight and body composition remains inconclusive.

Most studies have imposed calcium supplementation with either pills or dairy products; however, some consumers choose to supplement their calcium via calcium-fortified beverages. In support of this supplementation option, one of the more bioavailable forms of calcium, calcium citrate malate (CCM) [9], is available in commercially available calcium-fortified fruit juice. Previous research indicates that consumption of fruit juice fortified with CCM results in significantly greater calcium absorption than milk or calcium carbonate [9,10]. Thus, CCM may have a better likelihood of eliciting a metabolic effect. Limited, if any, data exist demonstrating the efficacy of calcium-fortified juices at attenuating fat accretion. Therefore, the purpose of this investigation was to determine the effectiveness of increasing the calcium:protein ratio using a beverage fortified with calcium (1,125 mg Ca^++^/d of CCM) at decreasing body weight and body fat in free-living postmenopausal women.

## Methods

### Study design and supplementation

The study was approved by the Kansas State University Institutional Review Board, and written informed consent was obtained from each subject. Subjects were recruited through newspaper advertisements, word of mouth, and flyers posted on the Kansas State University campus and in the surrounding community. Respondents were screened for eligibility during a telephone interview. Women were eligible for the study if they were between 45 and 75 years of age and postmenopausal. Menopause was defined as one year from the date of the last menstrual period, or the date of operation for surgical menopause. Exclusion criteria included current diagnosis of chronic disease; smoking; fracture within past year; beginning or ending hormone replacement therapy within six months; and taking any of the following medications known to affect bone metabolism: bisphosphonates, thiazides, corticosteroids, calcitonin, or tamoxifen.

Forty-one healthy postmenopausal women were enrolled, and 37 completed the study (age = 60 ± 7 yr; wt = 69.5 ± 9.9 kg; BMI = 26.1 ± 3.7; years post-menopause = 12.5 ± 9.6 yr). The subjects were randomly assigned, with stratification according to body composition, to either a supplement group (CAL, n = 17) or a free-living comparison group (CTL; n = 20). Subjects in the CAL group consumed 591 ml/day of a beverage containing milk (7%) and fruit juice (15%) (Cal-C™, Nutrijoy, Inc., Manhattan, KS). They were asked to consume half the beverage in the morning the remainder later in the day. Control subjects were asked to continue their usual lifestyle (diet and daily activities). Four subjects in the CAL group withdrew before completion of the study, two due to unwillingness to continue daily supplementation and two due to personal reasons not related to the study. Subjects (n = 2) who travelled during the year for an extended period (two to three weeks) were given the option to carry the beverages with them or take two calcium citrate pills (600 mg calcium per pill) per day in place of the beverage. Compliance was 97%, as measured by weekly consumption reports, which subjects turned in when they picked up their drinks each week. Reasons for missing included: gastrointestinal discomfort, forgetfulness, flu, family emergency, and surgery.

### Measurements

At baseline and after the intervention (POST), the subjects reported to the laboratory for height, weight, and dual-energy x-ray absorptiometry (DXA) measurements; to turn in 3-day diet records; and to complete the Yale Physical Activity Survey (YPAS) [11]. Total body composition (body fat percentage, %BF_TB_; fat mass, FM; and non-bone fat-free mass, FFM) was measured via DXA (v5.6, GE Lunar Corp., Milwaukee, WI). Abdominal percentage fat (%BF_Ab_) was determined by using a customized region of the abdomen (L2 to the iliac crest) as previously described [12]. The same technician analyzed baseline and POST assessments and was blinded to treatment. All subjects were given oral and written instructions for the completion of the diet records at baseline and 12 months. The 3-d diet records (2 weekdays and 1 weekend day) were analyzed using commercially available software (Nutritionist Pro™, v 2.0, First DatBank, Inc., San Bruno, CA). One CTL group subject did not complete a baseline diet record. Physical activity was estimated by using the YPAS [11], which yields an index score that has been associated with VO_2max _in the elderly and is related to energy expenditure measured via doubly labelled water [13].

### Statistical analyses

A general linear model analysis of variance with repeated measures (group by time) was used to determine interaction and main effects, and independent and paired *t*-tests were used to compare differences at each testing period. Significance was set at p < 0.05. All analyses were run using SPSS for Windows software (version 11.5; SPSS Inc., Chicago, IL). *Post hoc *Pearson product moment correlations were used to determine the relationship between nutrient intake, nutrient ratios, and changes in body composition. Statistical software (Axum, version 7.0, MathSoft, Cambridge, MA) was used to calculate statistical power. All values are presented as mean ± SD.

## Results

### Body composition

There were no differences between groups at baseline for any of the body-composition variables (Table 1). There was a main effect of time for body fat. At POST in the CTL group, the non-bone fat-free mass was decreased compared to baseline.

**Table 1:** Whole-body Body composition measures of participants at baseline and 12 months, with (CAL) and without (CTL) calcium supplementation.

	**Baseline**	**POST**	ρ
**Body Weight **(kg)			
CAL	69.6 ± 11.5	70.2 ± 12.6	0.5 ± 3.3
CTL	69.3 ± 8.7	69.3 ± 8.3	0.0 ± 2.9
**Whole-body fat **(%)			
CAL	43.0 ± 7.3	43.1 ± 6.7	0.0 ± 2.3
CTL	43.1 ± 5.5	43.1 ± 5.4	0.1 ± 2.4
**Abdominal fat **(%)			
CAL	45.3 ± 9.7	44.7 ± 8.7	-0.6 ± 3.4
CTL	44.0 ± 8.1	44.2 ± 8.3	0.2 ± 3.7
**Fat Mass **(kg)^#^			
CAL	29.3 ± 9.2	30.9 ± 9.9	1.6 ± 2.8
CTL	29.0 ± 7.1	30.1 ± 6.7	1.3 ± 2.7
**Fat-free Mass **(kg)			
CAL	39.1 ± 2.9	39.2 ± 2.7	0.1 ± 1.3
CTL	40.0 ± 3.7	39.6 ± 4.0*	-0.4 ± 0.7
**Bone Mineral Content **(kg)			
CAL	2.5 ± 0.3	2.5 ± 0.4	0.0 ± 0.1
CTL	2.4 ± 0.3	2.4 ± 0.3	0.0 ± 0.1

Mean ± SD; Abdominal fat = % fat from custom region of interest of the abdomen; 1475-2891-4-21-i1.gif"/> = absolute difference between POST and baseline; ^#^= main effect for time. * = within group difference from baseline to post.

### Dietary intake and physical activity

Total energy, carbohydrate, and calcium intakes, and the calcium:protein ratio (mg/g), increased significantly compared with baseline values in the CAL group (Table 2). At POST, dietary calcium and the calcium:protein ratio were greater in the CAL group than in the CTL group. There was also a difference in the delta values between groups for carbohydrate and calcium intakes and in the calcium:protein ratio. The CTL group did not experience any significant changes in dietary calcium intake over time. There was no difference between or within groups for reported physical activity, as measured by the YPAS index, at baseline (CAL = 40 ± 18; CTL = 51 ± 25) or POST (CAL = 40 ± 14; CTL = 49 ± 23).

**Table 2:** Dietary intake of total energy, macronutrients and calcium values as reported using 3-day diet records.

	**Baseline**	**POST**	ρ1475-2891-4-21-i1.gif"/>
**Energy Intake **(MJ/d)			
CAL	6.88 ± 1.28	7.89 ± 1.77^†^	1.0 ± 1.8
CTL	7.96 ± 2.50	7.96 ± 2.16	-0.0 ± 1.7
**Carbohydrate **(g/d)			
CAL	219 ± 40	270 ± 65^††^	51 ± 48*
CTL	239 ± 60	243 ± 69	1 ± 63
**Protein **(g/kg/d)			
CAL	0.97 ± 0.2	1.09 ± 0.5	0.1 ± 0.3
CTL	1.10 ± 0.3	1.07 ± 0.4	-0.0 ± 0.5
**Carbohydrate:Protein Ratio **(g/g/d)			
CAL	3.4 ± 0.8	3.9 ± 0.8	1.2 ± 0.4
CTL	3.3 ± 0.9	3.5 ± 1.2	1.1 ± 0.4
**Fat **(g/d)			
CAL	59 ± 20	62 ± 16	3 ± 18
CTL	72 ± 39	72 ± 34	0 ± 26
**Calcium **(mg/d)^‡#^			
CAL	919 ± 435	2201 ± 763** ^†^	1281 ± 824**
CTL	1244 ± 734	1153 ± 647	-90 ± 567
**Calcium:Protein Ratio **(mg:g/d)^‡#^			
CAL	14.6 ± 8.2	32.3 ± 15.6** ^† ^^†^	17.7 ± 15.8**
CTL	16.8 ± 8.7	15.2 ± 7.5	-1.0 ± 7.5
**Calcium:Energy **(mg:MJ/d)^‡#^			
CAL	134 ± 67	286 ± 105** ^† ^^†^	152 ± 108**
CTL	162 ± 93	144 ± 93	-18 ± 78

Mean ± SD; 1475-2891-4-21-i1.gif"/> = absolute difference between POST and baseline; 1475-2891-4-21-i2.gif"/> = fold change from baseline to POST; ^‡ ^= main effect for time; ^#^= main effect for diet; ^† ^*p *< 0.05, ^††^p < 0.001 different from zero months within group; * *p *< 0.05, ** *p *< 0.01, difference between CAL and CTL groups.

## Discussion

The primary finding of this study was that calcium supplementation (1,125 mg/d) did not decrease body weight or fat in the CAL group. The present results do not support the notion that increasing calcium intake leads to decreased body weight in apparently healthy older women.

The results from the present 12 month study differ from those reported by Zemel et al. [1]. The calcium supplement (600 mg Ca^++^/d) in the Zemel et al. study [1] was an unsweetened dairy product (yogurt), whereas the calcium from this study was suspended in fruit juice. Also, the men in the study by Zemel et al. [1] were obese and diagnosed with Type 2 diabetes; and, they consumed less than 500 mg Ca^++^/d, which is indicative of an unhealthy diet [14]. The women in the present study were apparently healthy, most were overweight or obese, and consumed almost twice the amount of calcium per day than the men in Zemel's study [1].

An explanation for a lack of change in body composition in the present study might be that levels of serum calcium and 1,25-dihydroxyvitamin D (1,25-(OH)_2 _D) did not change with the intervention as would likely occur with calcium deficient individuals. This is critical, as the transgenic animal model suggests that changes in serum calcium and 1,25-(OH)_2 _D are what drive the upregulation of lipolysis and inhibit lipogenesis in adipocytes via alterations in intracellular calcium concentrations and fatty acid synthesis mRNA expression [15]. In the dairy supplementation study by Gunther et al. [16] no changes were observed for 1,25-(OH)_2 _D or parathyroid hormone following increased calcium intake from dairy sources (1,000 – 1,400 mg Ca^++^/d).

Another explanation might be that the low carbohydrate:protein ratio of the dairy product played a role in the effectiveness of the dairy products to decrease body fat by 4.9 kg in 12 months. Layman et al. [17] have demonstrated that the carbohydrate:protein ratio plays an important role in weight loss, with a lower ratio being preferred. The macronutrient composition of the present calcium supplement may be important since the carbohydrate:protein ratio of milk is ~1.3 (12 g carbohydrate and 9 g of protein), whereas the ratio of the supplement used in this study was ~25 (25 g carbohydrate and <1 g protein per serving). However, this theory is not supported by results from Gunther et al. [16], which illustrate that one year of calcium supplementation, via dairy products, did not change body composition in young women. Thus, in healthy younger women, the use of a low carbohydrate:protein ratio means of supplementing calcium was not efficacious.

In support of the present data, it was noted in a review by Barr [18] that only one out of 17 studies reviewed observed decreased body weight during calcium supplementation. Moreover, in a short-term clinical trial by Barr et al. [8], healthy older adults were randomly assigned to control group or a group that consumed three servings of milk (skim or 1% fat) per day for 12 weeks. They reported a significant interaction effect (treatment by time) with the milk-consuming group gaining 0.6 kg more weight than the control group.

What was somewhat surprising was the amount of calcium habitually consumed by the women in the present study at baseline (1,112 ± 630 mg calcium/d), compared with the reported intakes of older women from other studies [19,20]; however, an upper limit or threshold of calcium intake has not been suggested relative to effects on body composition. This higher-than-expected calcium intake might reflect the socioeconomic and/or educational background [21] of the women in the present study, a factor we did not assess.

Also, even with a two-fold increase in the calcium:protein ratio there were no changes in any measure of body composition. Based on the prediction equation by Davies et al. [2], the CAL group should have lost 0.49 kg of body weight and the control group should have gained 0.14 kg. This expected decrease in body weight may have been prevented by the fact the CAL group reported a significant increase in energy intake at POST compared with baseline. However, it is difficult to know whether the 3-day diet records and the YPAS accurately reflect the energy intake and expenditure patterns over the 12 months of this study, or if the calcium-fortified beverage attenuated the expected decrease in body fat via the high carbohydrate:protein ratio of the drink. Regardless, these results illustrate that merely increasing the calcium:protein ratio alone is not enough to change body weight or body fat as predicted or suggested by previous epidemiological and animal studies.

The strengths and novelty of this randomized clinical study were: 1) the efficacious nature of the design; 2) the use of a non-dairy calcium-fortified beverage; 3) the use of the calcium:protein ratio as a dependent variable relative to changes in body composition; and 4) this was one of the longer calcium supplementation studies specifically reporting changes in abdominal adiposity. As stated by Barr [18], there is limited published data from randomized trials that have investigated the effect of calcium supplementation on body composition. Contrary to the epidemiological and animal studies, this study clearly demonstrates that a dramatic change in the calcium:protein does not ubiquitously decrease body weight, fat weight, or percent body fat over a 12 month period in apparently healthy free-living women as predicted. That said, given the reported increase in energy intake over three days, the increased calcium intake may have attenuated a potential increase in body weight and/or body fat. Future studies utilizing controlled diets to maintain consistent macronutrient intakes are needed to insure minimal changes in other nutrients occur and to establish whether effects observed in animal studies occur in healthy humans.

## Conclusion

In conclusion, 12 months of supplementation with a calcium-fortified beverage seems to have no effect on body composition in free-living postmenopausal women who already consume higher-than-expected levels of calcium. Thus, significantly increasing the calcium:protein ratio alone did not decrease body weight or body fat in these free-living postmenopausal women.

## Competing interests

The author(s) declare that they have no competing interests.

## Authors' contributions

TRS and MDH worked together to write the first draft of the manuscript. TRS was responsible for subject recruitment and baseline data collection. CMC, EKA, and MDH assisted with baseline data collection and performed data collection and analysis at the end of the study. VMR and CAH helped develop the research design and edited the manuscript.
